# Estradiol is significantly associated with prognosis in non-surgical liver cancer patients: from bench to bedside

**DOI:** 10.18632/aging.202280

**Published:** 2021-01-10

**Authors:** Rangrang Wang, Yuan Liu, Hongze Sun, Tao Wang, Changcan Li, Junwei Fan, Zhaowen Wang

**Affiliations:** 1 Department of General Surgery, Shanghai General Hospital, School of Medicine, Shanghai Jiao Tong University, Shanghai, China; 2 Department of General Surgery, Tongji Hospital, Tongji University School of Medicine, Shanghai, China

**Keywords:** liver cancer, non-surgical, estradiol, gender, SEER

## Abstract

There are rarely systematic studies to analyze the prognostic factors among non-surgical liver cancer patients. Whether there is a gender difference in the survival of non-surgical liver cancer patients and what may cause this difference is still unclear.

A total of 12,312 non-surgical liver cancer patients were enrolled in this study. Age, race, sex, grade, tumor TNM stage, marital status, tumor size, and histological type were independent risk factors in liver cancer and were confirmed in the validation cohort. Before menopause, females demonstrated a better mean survival probability than males (39.4±1.4 vs. 32.7±0.8 months, respectively; p<0.001), and continued in post-menopause. The results of differentially expressed genes (DEGs) and KEGG pathway analysis showed that there were significant differences in steroid hormone biosynthesis between male and female liver cancer patients. *In vitro* experiments revealed that estradiol inhibited the proliferation of hepatocellular cancer cell lines and increased apoptosis, but estrone exerted no effect.

In conclusion, gender differences in prognosis among non-surgical liver cancer patients were confirmed and attributable primarily to estradiol.

## INTRODUCTION

Liver cancer is the sixth most common cancer, and the fourth most common cause of cancer-related death reported in CA: A Cancer Journal for Clinicians [1], with an annual incidence of 841,080 patients [1]. It is estimated that about 42,810 individuals will be diagnosed with liver cancer in the United States in 2020, of whom 30,160 will die [2]. In virtually all countries, males exhibit incidence and mortality rates 2 to 3 times higher than women [1, 3]. As such, addressing the high mortality and poor prognosis associated with liver cancer remains an urgent priority.

In recent years, some studies have reported significant differences in survival rates between men and women among those with liver cancer [4–8]. Also, multivariate Cox regression analyses reveal that race [5–8], marriage [6], and economic status [9] also affect the survival of individuals with liver cancer. In contrast, however, some other studies have reported no significant prognostic differences between males and females [8]. Some recent studies have reported that estrogen may play a role in the prognosis of patients with liver cancer [10–12], while others have reported no such effect [3]. Unfortunately, most—if not all—of these studies were performed using a pool of patients in surgical and non-operative states, even though surgery is a vital factor influencing patient prognosis [13]. As such, they could not fully reveal the true impact of gender differences on the prognosis of liver cancer patients in natural states.

To reveal the real-world situation, our study focused on factors affecting prognosis in patients with liver cancer who had not undergone surgery and, in addition, we also performed related *in vitro* experiments. The results revealed a significant difference between males and females in terms of survival among those with liver cancer. The results of *in vitro* experiments revealed that, although estrone did not have a significant effect on liver cancer cells, estradiol played a central role.

## RESULTS

### Meta-analysis of the influence of gender on the OS of patients with liver cancer

The literature search retrieved 424 unique citations. After screening of titles and abstracts, 87 full-text articles remained for assessment, reporting that females had longer survival than males in different type cancer (pooled HR 0.86 [95% CI: 0.83–0.89]; p < 0.001; I^2^ = 98%) (Supplementary Figure 1). Finally, 6 studies including 98,010 individuals were reviewed to appraise the effect of gender on the OS of liver cancer patients. The forest plot of gender for the efficacy in reducing the risk for prognosis in liver cancer patients is shown in Supplementary Figure 2. Among patients with liver cancer, females demonstrated better prognosis than males (pooled HR 0.93 [95% CI: 0.91–0.94]; p < 0.001; I^2^ = 0%), and homogeneity performed well.

### Characteristics of liver cancer patients from the SEER database

Data from 12,312 liver cancer patients housed in the SEER research database, who had not undergone surgery between 2010 and 2015, were included in this study. All participants were randomly divided into the primary (70% [n = 8658]) or validation (30% [n = 3654]) cohort. The baseline characteristics of these cohorts are summarized in Table 1. Among all patients, 27.2% (n = 2375) were female and 72.8% (n = 8966) were male. In total, 17.9% (n = 2200) were ≤ 55 years of age at diagnosis and 82.1% (n = 10,112) were > 55 years of age. The distribution according to race/ethnicity was as follows: white, 69.5% (n = 8553); black, 12.8% (n = 1580); and other (American Indian/AK Native, Asian/Pacific Islander), 17.7% (n = 2179). According to the American Joint Committee on Cancer, 28.5% (n = 3515) of patients were grade I at diagnosis, 47.2% (n = 5809) were grade II, 22.7% (n = 2800) were grade III, and 1.5% (n = 188) were grade IV. Among these individuals, 57.6% (n = 7090) were married, 19.1% (n = 2348) were single, 11.2% (n = 1378) were divorced, and 10.3% (n = 1269) were widowed. According to pathology, 88.7% (n = 10,924) had HCC, 11.1% (n = 996) had intrahepatic cholangiocarcinoma (ICCs), and others were combined. Regarding tumor size, 26.1% (n = 3217) of individuals exhibited lesions < 30 mm and 73.9% (n = 9095) had lesions > 30 mm. Tumor TNM stages are summarized in Table 1. Percentages were similar between the primary and validation cohorts.

**Table 1:** Baseline characteristics of patients.

**Characteristic**	**All of patients(n=12312)**		**Primary cohort(n=8658)**		**Validation cohort(n=3654)**	
	**No of patients**	**%**	**No of patients**	**%**	**No of patients**	**%**
Age						
≤55	2200	17.9	1552	17.9	648	17.7
>55	10112	82.1	7106	82.1	3006	82.3
Race						
White	8553	69.5	6052	69.9	2501	68.4
Black	1580	12.8	1088	12.6	492	13.5
Other ^a^	2179	17.7	1518	17.5	661	18.1
Sex						
Male	8966	72.8	6283	72.6	2683	73.4
Female	3346	27.2	2375	27.4	971	26.6
Grade						
1	3515	28.5	2515	29.0	1000	27.4
2	5809	47.2	4039	46.7	1770	48.4
3	2800	22.7	1965	22.7	835	22.9
4	188	1.5	139	1.6	49	1.3
Tumor T						
T0	9	0.1	6	0.1	3	0.1
T1	5676	46.1	3991	46.1	1684	46.1
T2	3068	24.9	2166	25.0	902	24.7
T3	2772	22.5	1947	22.5	825	22.6
T4	505	4.1	354	4.1	151	4.1
TX	283	2.3	194	2.2	89	2.4
Tumor N						
N0	10640	86.4	7476	86.3	3164	86.6
N1	1062	8.6	763	8.8	299	8.2
NX	610	5.0	419	4.8	191	5.2
Tumor M						
M0	10855	88.2	7626	88.1	3229	88.4
M1	1457	11.8	1032	11.9	425	11.6
Marital status						
Divorced	1378	11.2	952	11.0	426	11.7
Single	2348	19.1	1639	18.9	709	19.4
Married	7090	57.6	5005	57.8	2085	57.1
Separated	185	1.5	134	1.5	51	1.4
Windowed	1269	10.3	899	10.4	370	10.1
Unmarried or Domestic partner	42	0.3	29	0.3	13	0.4
Tumor size (mm ^c^)						
≤30	3217	26.1	2265	26.2	952	26.1
>30	9095	73.9	6393	73.8	2702	73.9
Histologic Type						
ICCs	1367	11.1	996	11.5	371	10.2
HCC	10924	88.7	7648	88.3	3276	89.7
Combined ^b^	21	0.2	14	0.2	7	0.2

Abbreviations: HCC, hepatocellular carcinoma; ICCs, intrahepatic cholangiocarcinomas.

^a^ Contain American Indian/AK Native, Asian/Pacific Islander.

^b^ HCC+ICCs.

^c^ millimeter.

### Survival analysis

Univariate and multivariate Cox regression hazards models were used to analyze prognostic factors in patients with liver cancer (Tables 2, 3). In the univariate Cox models, age, sex, race, grade, tumor TNM stage, marital status, tumor size, and histological type were significantly associated with OS of non-surgical liver cancer patients (p < 0.05). Multivariate Cox analysis confirmed that all of those variables were independent prognostic factors for OS (p < 0.05).

**Table 2:** Univariate cox regression analyses of primary and validation cohorts.

**Characteristic**	**Primary cohorts(n=8658)**			**Validation cohorts(n=3654)**		
	**HR**	**95%CI**	**p value**	**HR**	**95%CI**	**p value**
Age			<0.001			<0.001
≤55	Ref			Ref		
> 55	1.165	(1.080-1.257)	<0.001	1.398	(1.239-1.576)	<0.001
Race			<0.001			<0.001
White	Ref			Ref		
Black	1.154	(1.061-1.255)	0.001	1.147	(1.012-1.300)	0.031
Other	0.818	(0.755-0.886)	<0.001	0.788	(0.699-0.889)	<0.001
Sex			0.001			0.047
Male	Ref			Ref		
Female	0.898	(0.842- 0.958)	0.001	0.903	(0.817-0.998)	0.047
Grade			<0.001			<0.001
1	Ref			Ref		
2	1.039	(0.969-1.115)	0.284	0.995	(0.894-1.107)	0.923
3	1.932	(1.789-2.087)	<0.001	1.644	(1.459-1.852)	<0.001
4	2.169	(1.764-2.666)	<0.001	2.115	(1.521-2.940)	<0.001
Tumor T			<0.001			<0.001
TX	Ref			Ref		
T0	1.537	(0.680-3.474)	0.301	0.936	(0.295-2.970)	0.910
T1	0.298	(0.253-0.351)	<0.001	0.312	(0.246-0.397)	<0.001
T2	0.354	(0.299-0.419)	<0.001	0.359	(0.280-0.459)	<0.001
T3	0.877	(0.744-1.033)	0.116	0.875	(0.688-1.113)	0.277
T4	0.861	(0.708-1.049)	0.137	0.929	(0.695-1.243)	0.620
Tumor N			<0.001			<0.001
N0	Ref			Ref		
N1	2.577	(2.360-2.813)	<0.001	2.412	(2.102-2.769)	<0.001
NX	2.273	(2.025-2.553)	<0.001	2.564	(2.175-3.022)	<0.001
Tumor M			<0.001			<0.001
M0	Ref			Ref		
M1	3.527	(3.270-3.804)	<0.001	3.219	(2.866-3.616)	<0.001
Marital status			<0.001			<0.001
Divorced	Ref			Ref		
Single	1.051	(0.946-1.169)	0.354	0.986	(0.844-1.152)	0.863
Married	0.855	(0.780-0.938)	0.001	0.795	(0.694-0.911)	0.001
Separated	1.141	(0.905-1.437)	0.264	1.237	(0.869-1.762)	0.238
Windowed	1.222	(1.087-1.374)	0.001	0.990	(0.828-1.183)	0.911
Unmarried or Domestic partner	0.515	(0.256-1.036)	0.063	0.622	(0.277-1.398)	0.251
Tumor size(mm)			<0.001			<0.001
≤30	Ref			Ref		
>30	2.382	(2.208-2.569)	<0.001	2.265	(2.020-2.541)	<0.001
Histologic Type			0.009			0.280
ICCs	Ref			Ref		
HCC	0.872	(0.798-0.952)	0.002	0.895	(0.776-1.033)	0.129
Combined	0.797	(0.378-1.679)	0.550	0.708	(0.263-1.903)	0.493

**Figure 1:**
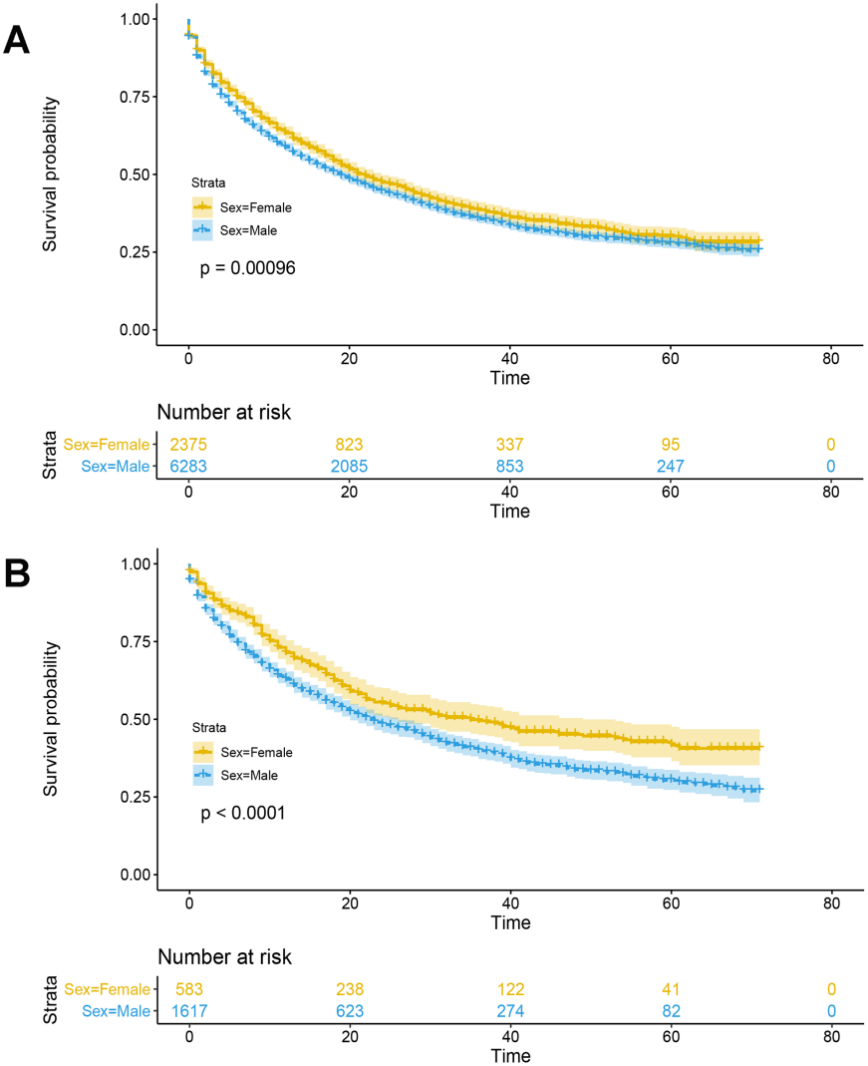
**Kaplan-Meier survival analysis with log-rank test was performed in non-surgical liver cancer patients.** (**A**) Kaplan-Meier survival analysis with log-rank test was performed in non-surgical liver cancer patients in the primary cohort. (**B**) Before menopause (i.e., ≤ 55 years of age), women with liver cancer have a better prognosis than men (p<0.0001, log-rank test).

According to the results of multivariate Cox analysis, patients < 55 years of age had a worse prognosis than those ≤ 55 years of age (HR 1.198 [95% CI: 1.109-1.295; p < 0.001) (Table 3). Compared with Caucasians, other races (i.e., American Indian/AK Native, Asian/Pacific Islander) had a better prognosis (HR 0.805 [95% CI: 0.742-0.873; p < 0.001), but blacks did not (p = 0.068) (Table 3). There were significant prognostic differences between males and females among non-surgical liver cancer patients. Females demonstrated a better prognosis than males (p < 0.001) (Figure 1A), and survived longer than males (32.3 ± 0.7 vs. 30.1 ± 0.4 months, respectively; p = 0.001). Compared with males, females had a lower hazard ratio (HR 0.868 [95% CI: 0.810-0.930; p < 0.001). As the clinical grade increased, patient prognosis worsened (p < 0.001); however, grade 2 was not an independent risk factor for prognosis in non-surgical liver cancer patients (p = 0.586). The HRs for tumor TNM stage are shown in Table 3. Patients who were married demonstrated better prognosis (HR 0.805 [95% CI: 0.805-0.953]; p = 0.011), and widowed patients demonstrated poor prognosis (HR 1.251 [95% CI: 1.029-1.410]; p < 0.001). Compared with ICCs, patients with HCC have a worse prognosis (HR 1.168 [95% CI: 1.060-1.287]; p = 0.002).

**Table 3:** Multivariate cox regression analyses of primary and validation cohorts.

**Characteristic**	**Primary cohort(n=8658)**			**Validation cohort(n=3654)**		
	**HR**	**95%CI**	**p value**	**HR**	**95%CI**	**p value**
Age			<0.001			<0.001
≤55	Ref			Ref		
> 55	1.198	(1.109-1.295)	<0.001	1.442	(1.275-1.630)	<0.001
Race			<0.001			0.033
White	Ref			Ref		
Black	1.084	(0.994-1.182)	0.068	1.056	(0.929-1.200)	0.404
Other	0.805	(0.742-0.873)	<0.001	0.863	(0.763-0.977)	0.020
Sex			<0.001			0.012
Male	Ref			Ref		
Female	0.868	(0.810-0.930)	<0.001	0.873	(0.784-0.971)	0.012
Grade			<0.001			<0.001
1	Ref			Ref		
2	0.980	(0.913-1.053)	0.586	0.974	(0.874-1.085)	0.627
3	1.578	(1.456-1.710)	<0.001	1.442	(1.276-1.631)	<0.001
4	1.615	(1.311-1.990)	<0.001	1.856	(1.327-2.595)	<0.001
Tumor T			<0.001			<0.001
TX	Ref			Ref		
T0	1.722	(0.754-3.932)	0.197	1.074	(0.332-3.477)	0.905
T1	0.489	(0.410-0.582)	<0.001	0.499	(0.385-0.645)	<0.001
T2	0.554	(0.463-0.663)	<0.001	0.564	(0.433-0.734)	<0.001
T3	1.014	(0.853-1.206)	0.874	1.026	(0.794-1.325)	0.846
T4	0.907	(0.739-1.113)	0.349	1.026	(0.756-1.392)	0.871
Tumor N			<0.001			<0.001
N0	Ref			Ref		
N1	1.447	(1.311-1.597)	<0.001	1.529	(1.312-1.783)	<0.001
NX	1.431	(1.264-1.621)	<0.001	1.790	(1.500-2.136)	<0.001
Tumor M			<0.001			<0.001
M0	Ref			Ref		
M1	2.190	(2.013-2.383)	<0.001	2.038	(1.793-2.317)	<0.001
Marital status			<0.001			<0.001
Divorced	Ref			Ref		
Single	1.060	(0.952-1.198)	0.288	1.016	(0.868-1.189)	0.845
Married	0.805	(0.805-0.953)	0.011	0.797	(0.694-0.915)	0.001
Separated	1.221	(0.969-1.540)	0.091	1.411	(0.990-2.011)	0.057
Windowed	1.251	(1.029-1.410)	<0.001	1.042	(0.867-1.252)	0.659
Unmarried or Domestic partner	0.547	(0.272-1.101)	0.091	0.572	(0.253-1.286)	0.177
Tumor size			<0.001			<0.001
≤30	Ref			Ref		
>30	1.752	(1.617-1.899)	<0.001	1.657	(1.466-1.873)	<0.001
Histologic Type			0.005			0.038
ICCs	Ref			Ref		
HCC	1.168	(1.060-1.287)	0.002	1.2220	(1.042-1.429)	0.013
Combined	0.802	(0.380-1.692)	0.563	0.862	(0.318-2.335)	0.771

### Prognostic nomogram for OS and ROC of the model

**Figure 2:**
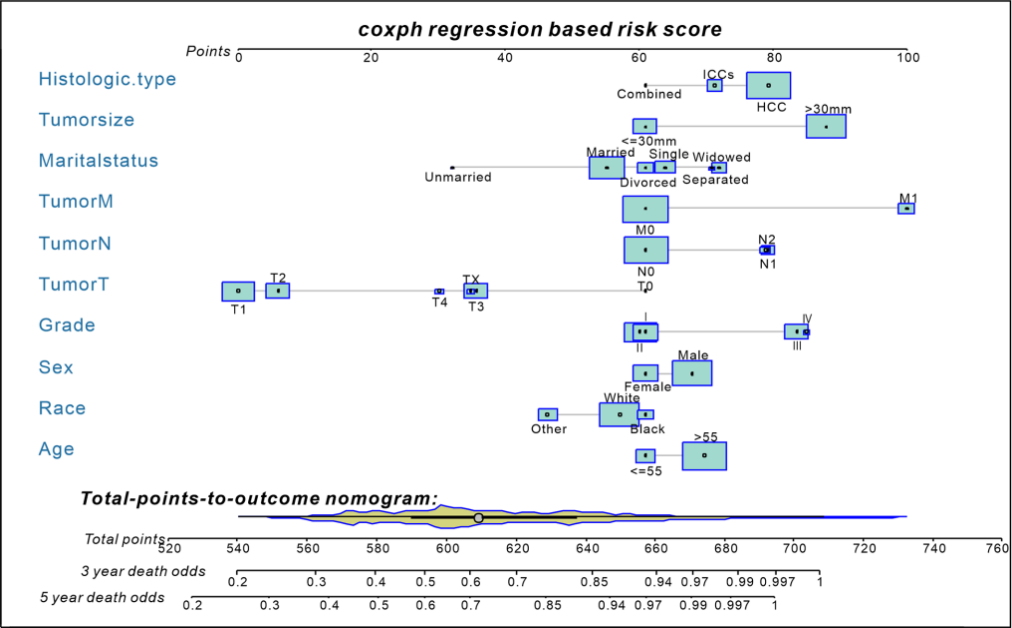
**A nomogram for predict 3- and 5-year death odds of non-surgical liver cancer patients (established by Cox regression model).** The yellow Violin Plot and the light blue box display the distribution of patients in the primary cohort. The size of the light blue box represents the proportion of patients. Abbreviations: HCC, hepatocellular carcinoma; ICCs, intrahepatic cholangiocarcinomas; Combined=HCC+ICCs.

**Figure 3:**
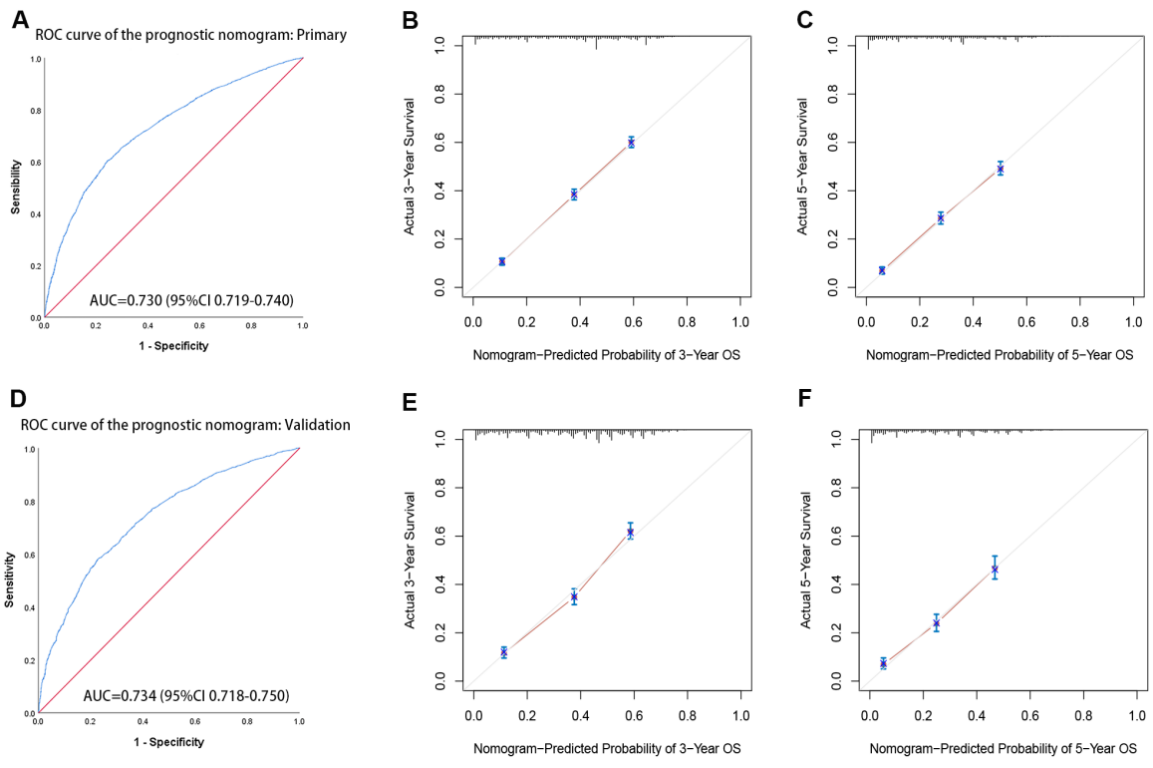
**The receiver operating characteristic (ROC) curves and calibration curve of the prognostic nomogram.** (**A**) ROC curves of the prognostic nomogram in the primary cohort. (**B**, **C**) The calibration curve of the nomogram-predicted probability in the primary cohort (3-year OS and 5-year OS, respectively). (**D**) ROC curves of the prognostic nomogram in the validation cohort. (**E**, **F**) The calibration curve of the nomogram-predicted probability in the validation cohort (3-year OS and 5-year OS, respectively). Age, sex, race, grade, tumor TNM stage, marital status, tumor size, and histological type are pooled in the primary(a) and validation(b) cohorts. AUC= area under the curve.

Based on independent prognostic factors identified in the multivariate Cox regression analysis, a prognostic nomogram was developed for liver cancer patients to predict 3-and 5-year survival probabilities (Figure 2). In primary cohorts, the area under the ROC curves (AUROCs) of the model was 0.730 (p < 0.001) (Figure 3A), the consistency index of the model was 71.56 (95%CI: 71.17-71.96), and the internal calibration curve for the probability of survival at 3-and 5-years showed a good agreement between the nomogram-predicted probability of OS and actual survival (Figure 3B, 3C). In the validation cohort, AUROCs were 0.734 (p < 0.001) (Figure 3D), the consistency index was 70.74 (95%CI: 70.13-71.35), there was also a well-performed calibration curve for survival prediction (Figure 3E, 3F). Therefore, the nomogram could reliably predict the 3- and 5-year OS probabilities. Furthermore, independent nomograms were developed for patients ≤ 55 and > 55 years of age (Supplementary Figures 3, 4).

### Survival differences according to sex and sex hormones: from bench to bedside

**Figure 4:**
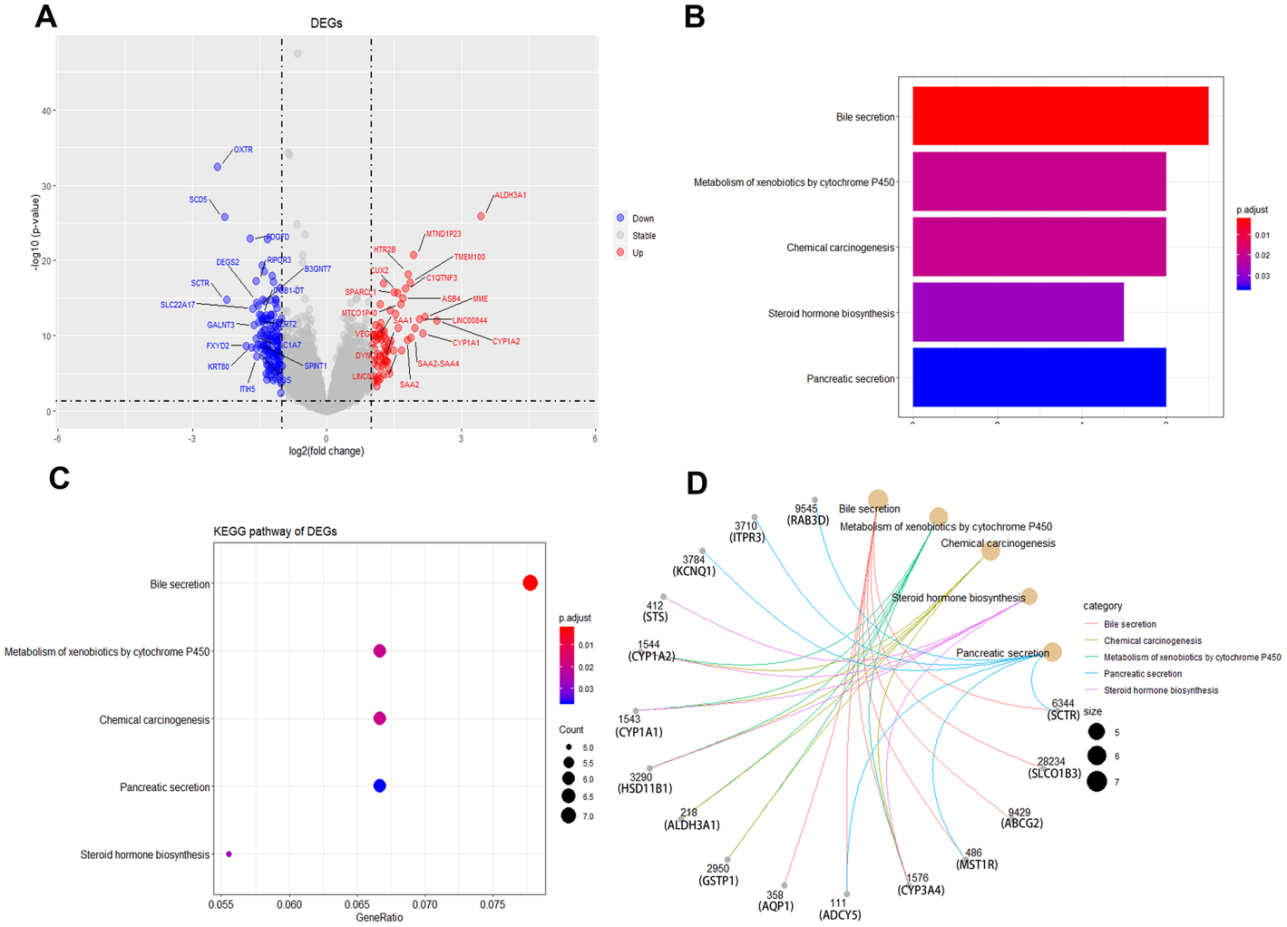
**Identification of differentially expressed genes and KEGG enrichment between male and female liver cancer patients.** (**A**) Volcano plot of male and female liver cancer patients in differentially expressed genes (DEGs). (**B**, **C**) KEGG enrichment of DEGs. (**D**) gene network diagram between DEGs and KEGG pathways.

The primary hypothesis, that female liver cancer patients have a better prognosis than males is attributable to different estrogen levels, was investigated. Based on previous studies [5, 14, 15], 55 years of age was defined as a surrogate for menopause. As shown in Figure 1B, before menopause (i.e., ≤ 55 years of age), there was an extreme gap between females and males in survival probability (39.4 ± 1.4 vs. 32.7 ± 0.8 months, respectively; p < 0.001). While this gap will be smaller after menopause (i.e., age > 55 years), the difference between females and males (30.5 ± 0.6 vs. 29.3 ± 0.4 months, respectively; p = 0.022) (Supplementary Figure 5) persisted. For further exploration, we obtained 265 male and 139 female liver cancer patients from the TCGA database, differentially expressed genes (DEGs) and KEGG pathway analysis results showed that there were significant differences in steroid hormone biosynthesis between male and female liver cancer patients (Figure 4).

**Figure 5:**
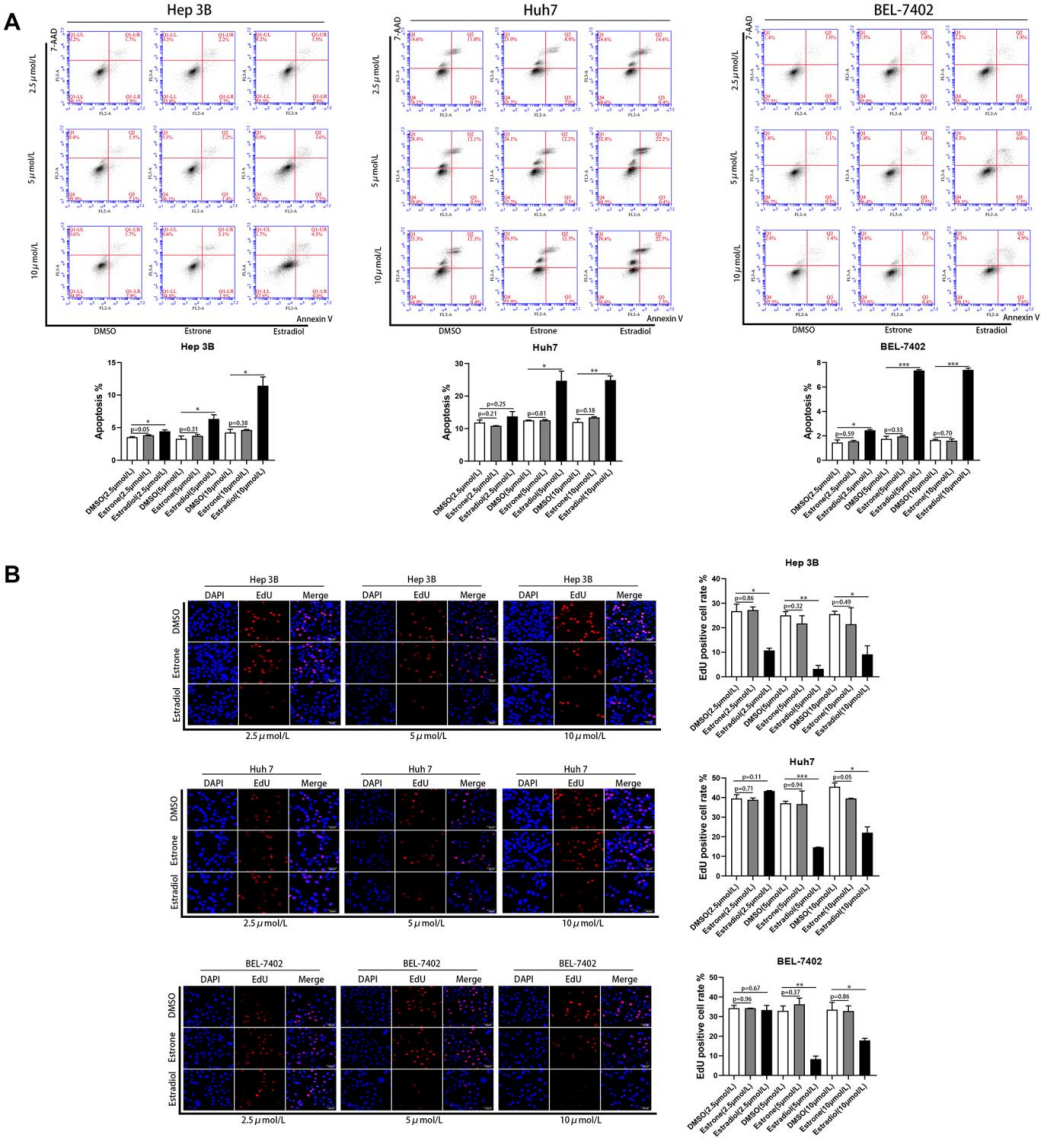
**Estradiol inhibited the proliferation and increased the apoptosis of liver cancer cell lines.** (**A**) Quantification of the apoptotic HCC cells population by flow cytometry. (**B**) Cell proliferation in HCC cells with different treatments was assessed using Cell-Light EdU Apollo 567 (catalog no· C10310-1; RiboBio). EdU positive cell rates were calculated; scale bar 50μm. Control (DMSO). *p<0.05, **p < 0·01, ***p<0·001. P < 0·05 was considered statistically significant. P values were calculated with Student's t-test.

According to a previous study [16], estradiol may be the main source of endogenous estrogen in postmenopausal women. This indicates that gender differences in patient prognosis may be primarily attributable to estrogen in patients with non-surgical liver cancer. To confirm the effect of estrogen on liver cancer, different concentrations of estrone and estradiol were used to stimulate HCC cell lines, including Hep 3B, BEL-7402, and Huh7. The results demonstrated that estradiol inhibited the proliferation of HCC cell lines and increased apoptosis; however, estrone exerted no effect (Figure 5). The most obvious effect was obtained when the concentration of estradiol is 5μmol/L. This result suggests that the difference in prognosis between men and women among liver cancer patients may be mainly attributed to estradiol.

## DISCUSSION

Although hepatic resection or liver transplantation is the optimal treatment for liver cancer [17], most patients with liver cancer are diagnosed at an intermediate-to-advanced stage when surgery is not suitable [18]. The proportion of surgical candidates is 5–10% due to multi-centric tumors, portal hypertension, vascular invasion, and dissemination [17]. Therefore, it is particularly important to design treatment plans for patients with liver cancer who cannot undergo surgery. Previous studies have hypothesized that women will benefit from estrones in liver cancer pathogenesis [11], epidemiological studies show that oophorectomy can increase the risk of liver cancer risk [19]. Interestingly, our study found that the gender differences in the prognosis of liver cancer patients always existed regardless of whether they were pre-menopausal (age ≤ 55 years) or post-menopausal (age > 55 years). Especially before menopause, there was a huge difference in survival between men and women but narrowed after menopause. Previous researches have shown that postmenopausal hormone replacement therapy is a protective factor in liver cancer [20, 21], and consistent with animal studies [20]. It means that as estrogen levels dropped, women’s benefits from hormones decreased and the dropped estrogen levels made the survival difference between men and women narrowed, and the benefits can be rescued by use postmenopausal hormone replacement therapy [20]. It reminds us that gender differences in the prognosis of liver cancer patients may be primarily attributable to estrogen [22].

In this study, meta-analysis revealed that females with liver cancer would live longer than males, which was consistent with the multivariate Cox regression analyses of the SEER database. The SEER database reported that age ≤ 55 years, white race, female sex, clinical grade 1, married status, and tumor size ≤ 30 mm were factors for a good prognosis in non-surgical liver cancer patients. We developed a prognostic nomogram for non-surgical liver cancer patients to predict 3-and 5-year OS rates, the AUROCs for this model was 0.730, and calibration curve performed well. Also, we found an extreme gap between males and females in terms of survival probability before menopause (age ≤ 55 years), and this gap persisted after menopause (age > 55 years). And we found that there was a significant difference in steroid hormone biosynthesis between male and female liver cancer patients. More importantly, we demonstrated that gender difference in the prognosis of liver cancer patients may be attributable to estradiol, but not estrone.

Previous research has suggested that men are more likely to experience liver cancer and have a worse prognosis than women [1, 2]; however, other studies have drawn contradictory conclusions [8]. Unfortunately, before our study, no meta-analysis had been performed to determine the association between sex and liver cancer risk. To confirm the influence of gender on the prognosis of liver cancer patients and eliminate bias, we analyzed previous studies and conducted a meta-analysis. The results revealed that female liver cancer patients would live longer than male patients (n = 98,010; p < 0.001). Our findings provide some epidemiological support that female liver cancer patients have a better prognosis than males with the disease.

Although some previous retrospective studies had investigated factors affecting the prognosis of liver cancer [6, 23], it is worth noting that all of them were performed using mixed cohorts of non-surgical and surgical patients, despite that is a vital factor influencing patient prognosis [13]. To eliminate confounding by surgery, we conducted a retrospective study using data from 12,312 liver cancer patients who had not undergone surgery between 2010 and 2015 from the SEER database. Multivariate Cox analysis revealed that under non-surgical conditions, the prognosis of female liver cancer patients is still better than that of males, which was consistent with the results of the meta-analysis. It is the largest retrospective study of non-surgical liver cancer patients, to our knowledge. Therefore, we can be relatively certain that gender has an impact on the prognosis of liver cancer patients and, perhaps, resolve the dispute in this regard.

We found that higher TNM stage and clinical-grade were associated with poor survival in liver cancer patients, as reported previously [24]. Tumor size remains an independent prognostic factor for liver cancer, and these results confirmed that patients with small HCC are essentially a heterogeneous group [25]. Among patients with liver cancer, those with white race have a better prognosis, consistent with the study by Zhang et al. [25]. Age, marital status, and histological type were all independent risk factors.

Nomograms have been widely used for cancer prognosis and displayed more accurate than conventional staging systems in the aspect of predicting prognosis in some cancers [26, 27]. Therefore, we conducted a prognostic nomogram for patients who had not undergone surgery. It is worth noting that this model had the most comprehensive index to predict the survival probability of non-surgical liver cancer patients at 3 and 5 years. The nomogram performed well, and its prediction was supported by the AUROCs (0.730 and 0.734 for the primary and validation cohorts, respectively). It was consistent with the observations by Zhang et al. [8], but they had too little data to find more valuable information.

Previous study supposed that estradiol is the main source of endogenous estrogen in postmenopausal women [16]. Whether the gender differences in the prognosis of liver cancer are attributable to estrogen still has not been resolved clearly. Unfortunately, previous studies focused only on the superficial aspects of retrospective research. But in our study, we used meta-analysis, cohort research, *in vitro* experiments, etc. to confirm our results. We proved that estradiol may, in large part, explain gender differences in the prognosis of liver cancer patients, and demonstrated that estradiol—but not estrone—inhibited the development of liver cancer, from bench to bedside. Because estradiol can inhibit tumor growth and increase tumor cell apoptosis rate, so as a protective factor, women may benefit from estradiol [28] and got longer survival than men. Our research provides the possibility of using hormone replacement therapy for liver cancer patients, which is supported by Zhong et al. [10] and McGlynn KA et. al [29].

In conclusion, we determined that gender differences in prognosis of liver cancer patients and the effect of sex hormones on the disease by used meta-analysis, retrospective analysis, and *in vitro* experiments. More importantly, we demonstrated that estradiol inhibited the proliferation of HCC cell lines and increased apoptosis, but estrone exerted no effect. This may explain why there was a gender difference in the prognosis of liver cancer patients. Future investigations should aim to elucidate the mechanisms of action of estradiol in those with liver cancer.

## MATERIALS AND METHODS

### Search strategy and selection criteria

A literature search of the PubMed database, performed using a combination of the keywords ((gender OR sex) AND “Marital status” AND race AND (cancer OR tumor)), retrieved a total of 424 relevant studies. After reviewing abstracts, 337 articles were excluded, and 87 tumor-related articles were used to verify the relationship between gender and tumor prognosis. Among these, 7 studies investigating liver cancer were retained, and one addressed liver transplantation. Ultimately, 6 studies were used to identify and appraise the effect of sex on overall survival (OS) in patients with liver cancer.

### Data sources and patient selection

The Surveillance, Epidemiology, and End Results (SEER) Program provides information regarding cancer statistics of the United States. SEER is supported by the Surveillance Research Program (SRP) in the National Cancer Institute’s Division of Cancer Control and Population Sciences (DCCPS) (https://seer.cancer.gov/). Data were based on Incidence-SEER 18 Regs Research Data + Hurricane Katrina Impacted Louisiana Cases (1973-2015), among which 123,806 liver cancer patients were screened. Excluding operable (n = 7188) and unknown (n = 118,682) liver cancer patients, data from 12,312 non-surgical liver cancer patients were analyzed using SEER*Stat software. 265 male and 139 female liver cancer patients were obtained from the TCGA database for differentially expressed genes (DEGs) and KEGG pathway analysis.

### *In vitro* experiments

The HCC cell lines Huh 7, Hep 3B, and BEL-7402 were maintained at 37° C in a humidified incubator with a 5% CO_2_ atmosphere. After treatment with different concentrations of estrone, estradiol, and dimethyl-sulfoxide (as control) for 24 h, cell apoptosis and proliferation experiments were conducted. Apoptosis experiments were performed as previously described [30], and flow cytometry was performed using a commercially available kit (Annexin V PE apoptosis kit, BD Biosciences, San Jose, CA, USA). Cell proliferation was assessed using Cell-Light EdU Apollo 567 (catalog no. C10310-1; RiboBio, China), as previously described [31].

### Statistical analyses

Statistical analyses were conducted using SPSS version 25.0 (IBM Corporation, Armonk, NY, USA), GraphPad Prism 8 (GraphPad Inc, San Diego, CA, USA) and the package of meta, ggplot, survival, and rms of R version 3.6.1.

Meta-analysis was performed using the package meta in R. The 95% confidence intervals (CI) reported in the available articles were extracted and pooled after calculating TE, seTE, and log transformed. Study heterogeneity was assessed using the I^2^ statistic [32]. Survival curves for both the genders were plotted using the Kaplan-Meier method by R. Univariate and multivariate Cox regression analyses were used to estimate independent risk factors in non-surgical liver cancer patients on OS and to generate hazard ratios (HR) and corresponding 95% CI in SPSS version 25. All results were verified in the validation cohort. A prognostic nomogram was developed based on multivariate Cox regression analyses. The receiver operating characteristic (ROC) curve was plotted using SPSS version 25. All statistical tests were two-tailed and differences with p < 0.05 were considered as statistically significant.

## Supplementary Material

Supplementary Figures
