# Supplementary material for: Estradiol is significantly associated with prognosis in non-surgical liver cancer patients: from bench to bedside
Source: Aging (Albany NY). 2021 Jan 10;13(3):3483–500. doi: 10.18632/aging.202280 (PMC7906196; doi:10.18632/aging.202280)
Supplement: Supplementary Figures [file aging-13-202280-s001.pdf]

SUPPLEMENTARY FIGURES

Study	TE	seTE	HR	95%-CI	Weight (fixed)	Weight (random)
Ailawadhi, S.,2012,Multiple myeloma (MM)	−0.10	0.0130	0.90	[0.88; 0.93]	2.9%	1.4%
Ailawadhi, S.,2014,Waldenstrom's macroglobulinemia	−0.18	0.0569	0.84	[0.75; 0.94]	0.1%	1.3%
Zhao, H. Y.,2019,BCLM(breast cancer liver metastases)	0.04	0.3548	1.04	[0.52; 2.08]	0.0%	0.2%
Zhang, Z. Y.,2016,Colorectal cancer	−0.05	0.0172	0.95	[0.92; 0.98]	1.6%	1.4%
Zhang, G.,2018,(ccRCC)clear cell renal cell carcinoma	−0.31	0.0343	0.74	[0.69; 0.79]	0.4%	1.4%
Zandberg, D. P.,2018,HNSCC(squamous cell carcinoma of the head and neck)	0.21	0.0597	1.23	[1.09; 1.38]	0.1%	1.3%
Yang, Q.,2019,LCNEC(pulmonary large cell neuroendocrine carcinoma)	−0.11	0.0485	0.89	[0.81; 0.98]	0.2%	1.3%
Yang, Q.,2019,ONSCLC(other non−small cell lung cancer)	−0.21	0.0050	0.81	[0.80; 0.82]	19.1%	1.5%
Yang, H.,2018,IA NSCLC(non−small−cell lung cancer)	−0.37	0.0485	0.69	[0.63; 0.76]	0.2%	1.3%
Yan, B.,2009,Colorectal Cancer	−0.25	0.0297	0.78	[0.74; 0.83]	0.5%	1.4%
Xu, C.,2017,Nasopharyngeal carcinoma	−0.11	0.0398	0.90	[0.83; 0.97]	0.3%	1.4%
Xie, Y.,2018,Tongue cancer	−0.10	0.0641	0.90	[0.80; 1.03]	0.1%	1.2%
Xie, J. C.,2018,Astrocytoma	−0.09	0.0114	0.92	[0.89; 0.94]	3.7%	1.5%
Xiao, Z.,2019,Hepatocellular carcinoma	−0.10	0.0301	0.91	[0.86; 0.96]	0.5%	1.4%
Wu, W.,2018,Hepatocellular carcinoma	−0.11	0.0254	0.90	[0.86; 0.95]	0.8%	1.4%
Wang, Q.,2019,Anal adenocarcinoma (AA)&rectal adenocarcinoma (RA)	0.07	0.2235	1.07	[0.69; 1.67]	0.0%	0.4%
Wang, F.,2019,Primary myelodysplastic syndrome	−0.22	0.0143	0.80	[0.78; 0.83]	2.4%	1.4%
Wang, B.,2019,Lung cancer bone metastasis	−0.12	0.0234	0.88	[0.84; 0.92]	0.9%	1.4%
Walker, G. V.,2014,one of the 10 most deadly cancers	−0.23	0.0100	0.79	[0.78; 0.81]	4.8%	1.5%
Wachtel, M. S.,2014,Glioblastoma	−0.06	0.0189	0.94	[0.91; 0.98]	1.4%	1.4%
Vest, M. T.,2012,Stage IIIA non−small cell lung cancer (NSCLC)	0.42	0.1355	1.52	[1.17; 1.98]	0.0%	0.8%
Tramontano, A. C.,2018,Esophageal Cancer	−0.62	0.0617	0.54	[0.48; 0.61]	0.1%	1.2%
Tchelebi, L.,2016,Rectal melanoma	0.19	0.2925	1.21	[0.68; 2.14]	0.0%	0.3%
Smith, E. C.,2012,Hodgkinlymphoma	−0.45	0.0516	0.64	[0.58; 0.70]	0.2%	1.3%
Singh, G. K.,2001,All−cause	−0.73	0.0127	0.48	[0.47; 0.49]	3.0%	1.4%
Shapiro, M.,2015,Pancreatic Cancer	−0.09	0.0473	0.92	[0.84; 1.01]	0.2%	1.3%
Seo, M.,2018,Non–muscle−invasive Bladder Cancer	−0.04	0.0159	0.96	[0.93; 0.99]	1.9%	1.4%
Saad, A. M.,2018,stage IV esophageal carcinoma	−0.03	0.0376	0.97	[0.90; 1.04]	0.3%	1.4%
Roche, A. M.,2016,Medullary Thyroid Cancer	0.14	0.1752	1.15	[0.82; 1.62]	0.0%	0.6%
Reyes, M. E.,2016,Colorectal cancer	0.44	0.0672	1.55	[1.36; 1.77]	0.1%	1.2%
Pan, Y.,2018,Primary spinal chordoma	−0.17	0.1037	0.85	[0.69; 1.04]	0.0%	1.0%
Pan, X. X.,2019,Nasopharyngeal carcinoma	−0.20	0.0653	0.82	[0.72; 0.93]	0.1%	1.2%
Pagedar, N. A.,2018,Head and neck cancer	0.07	0.0164	1.07	[1.04; 1.11]	1.8%	1.4%
Osazuwa−Peters, N.,2019,Head and neck cancer	−0.11	0.1470	0.90	[0.67; 1.20]	0.0%	0.7%
Olarte, L. S.,2014,Major Salivary Gland Cancer	−0.32	0.0407	0.72	[0.67; 0.79]	0.3%	1.4%
Niu, Q.,2018,Bladder urothelial carcinoma	−0.04	0.0146	0.96	[0.93; 0.99]	2.3%	1.4%
Nipp, R.,2018,Pancreatic cancer ...−...	0.03	0.0210	1.03	[0.99; 1.07]	1.1%	1.4%
Nipp, R.,2018,Pancreatic cancer stage...	−0.03	0.0197	0.97	[0.93; 1.01]	1.2%	1.4%
Nguyen, H. S.,2018,Subependymal Giant Cell Astrocytoma	0.22	0.5084	1.25	[0.46; 3.39]	0.0%	0.1%
Mukkamalla, S. K. R.,2018,Cholangiocarcinoma	−0.06	0.0136	0.94	[0.91; 0.96]	2.6%	1.4%
Mishra, M. V.,2011,Primary pancreatic lymphoma	−0.36	0.1510	0.70	[0.52; 0.94]	0.0%	0.7%
Menis, M.,2006,Colorectal cancer	0.06	0.0835	1.06	[0.90; 1.25]	0.1%	1.1%
Megwalu, U. C.,2017,Major Salivary Gland Adenoid Cystic Carcinoma	−0.01	0.2060	0.99	[0.66; 1.48]	0.0%	0.5%
Megwalu, U. C.,2014,Laryngeal Cancer	−0.21	0.0471	0.81	[0.74; 0.89]	0.2%	1.3%
Megwalu, U. C.,2016,Papillary thyroid microcarcinoma	−0.33	0.0601	0.72	[0.64; 0.81]	0.1%	1.2%
Megwalu, U. C.,2017,Oropharyngeal cancer	0.04	0.0393	1.04	[0.96; 1.12]	0.3%	1.4%
Megwalu, U. C.,2017, Oropharyngeal Cancer	0.04	0.0416	1.04	[0.96; 1.13]	0.3%	1.4%
Meguid, R. A.,2008,Colon cancers	−0.21	0.0119	0.81	[0.80; 0.83]	3.4%	1.5%
Liu, Y.,2019,Esophageal cancer	0.17	0.1327	1.19	[0.91; 1.54]	0.0%	0.8%
Liu, S.,2019,Anaplastic oligodendroglioma	−0.09	0.0624	0.92	[0.81; 1.04]	0.1%	1.2%
Liu, G.,2019,Papillary thyroid cancer	−0.65	0.0679	0.52	[0.46; 0.60]	0.1%	1.2%
Lin, D.,2018,Anal cancer	−0.48	0.0396	0.62	[0.57; 0.67]	0.3%	1.4%
Li, G.,2019,Pancreatic neuroendocrine tumor	−0.28	1.0205	0.76	[0.10; 5.58]	0.0%	0.0%
Klaassen, Z.,2016,Adrenocortical carcinoma	−0.16	0.0820	0.86	[0.73; 1.00]	0.1%	1.1%
Kim, J. A.,2013,Merkel Cell Carcinoma	−0.60	0.1375	0.55	[0.42; 0.72]	0.0%	0.8%
Kim, E.,2016,Anal Carcinoma	−0.47	0.0352	0.62	[0.58; 0.67]	0.4%	1.4%
Kim, A.,2017,Esophageal cancer	−0.15	0.0209	0.86	[0.83; 0.90]	1.1%	1.4%
Kamel, M. G.,2017,Non−Hodgkin's lymphoma(5years suvival)	−0.49	0.0335	0.61	[0.57; 0.65]	0.4%	1.4%
Kamel, M. G.,2017,Non−Hodgkin's lymphoma(10years suvival)	−0.47	0.0280	0.63	[0.59; 0.66]	0.6%	1.4%
Huang, J. F.,2019, Primary Spinal Chordoma	0.02	0.1586	1.02	[0.75; 1.39]	0.0%	0.7%
Hu, C. Y.,2019,Lip squamous cell carcinoma	−0.22	0.0797	0.80	[0.68; 0.94]	0.1%	1.1%
Horton, J. K.,2011,Soft tissue sarcoma	−0.40	0.1173	0.67	[0.53; 0.84]	0.0%	0.9%
Hall, M. D.,2015, Rectal Cancer	−0.31	0.0663	0.73	[0.64; 0.83]	0.1%	1.2%
Greenstein, A. J.,2007,Esophageal cancer	−0.06	0.1024	0.94	[0.77; 1.15]	0.0%	1.0%
Gore, J. L.,2005,Bladder carcinoma	0.09	0.0444	1.09	[1.00; 1.19]	0.2%	1.3%
Gleason, M. X.,2013,Pancreatic ductal adenocarcinoma	−0.12	0.0326	0.88	[0.83; 0.94]	0.5%	1.4%
Gleason, M. X.,2013,EPDAC (extended pancreatic ductal adenocarcinoma) survival	−0.10	0.0208	0.90	[0.87; 0.94]	1.1%	1.4%
Giri, S.,2015,Hairy Cell Leukemia	−0.14	0.0784	0.87	[0.75; 1.01]	0.1%	1.1%
Ellington, C. L.,2012,Adenoid cystic carcinoma of the head and neck	−0.31	0.0593	0.73	[0.65; 0.82]	0.1%	1.3%
Cronin, D. P.,2005,Non−Hodgkin's lymphoma(Localized and Regional Stage Lymphomas)	−0.59	0.2883	0.56	[0.32; 0.98]	0.0%	0.3%
Cronin, D. P.,2005,Non−Hodgkin's lymphoma(Distant Stage Lymphomas)	−0.18	0.2408	0.83	[0.52; 1.34]	0.0%	0.4%
Costa, L. J.,2016,Multiple myeloma	−0.11	0.0367	0.89	[0.83; 0.96]	0.4%	1.4%
Coburn, N. G.,2006,Gastric cancer	−0.20	0.0269	0.82	[0.77; 0.86]	0.7%	1.4%
Best, B.,2016,Head and Neck Cancer	−0.25	0.1768	0.78	[0.55; 1.10]	0.0%	0.6%
Chen, C.,2019,Acute myeloid leukemia	−0.05	0.0129	0.95	[0.92; 0.97]	2.9%	1.4%
Carroll, J. E.,2010,Pancreatic cancer	−0.10	0.0513	0.91	[0.82; 1.01]	0.2%	1.3%
Chavez−MacGregor, M.,2011,Breast cancer	−0.45	0.1828	0.64	[0.45; 0.91]	0.0%	0.6%
Borate, U. M.,2015,Acute myeloid leukemia	−0.14	0.0380	0.87	[0.81; 0.94]	0.3%	1.4%
Baine, M.,2011,Pancreatic cancer	−0.03	0.0105	0.97	[0.95; 0.99]	4.4%	1.5%
Amin, R. N.,2016, Esophageal Cancer	−0.07	0.4426	0.94	[0.39; 2.23]	0.0%	0.1%
Alshareef, S. H.,2019, Colorectal Cancer	−0.14	0.0263	0.87	[0.83; 0.92]	0.7%	1.4%
Al−Husseini, M. J.,2018,Colorectal cancer	−0.20	0.0053	0.82	[0.81; 0.82]	17.1%	1.5%
Al−Husseini, M. J.,2018,Glioblastoma	−0.06	0.0103	0.94	[0.92; 0.96]	4.6%	1.5%
Li Zhang,2019,Hepatocellular carcinoma	−0.10	0.0852	0.91	[0.77; 1.07]	0.1%	1.1%
Zhiyu Xiao,2019,Hepatocellular carcinoma	−0.10	0.0295	0.91	[0.86; 0.96]	0.6%	1.4%
Anthony S. Robbins,2008,Liver Transplantation Hepatocellular Carcinoma	0.11	0.1688	1.12	[0.80; 1.56]	0.0%	0.6%
Dongyun Yang, PhD,2014,Hepatocellular Carcinoma	−0.07	0.0136	0.93	[0.91; 0.96]	2.6%	1.4%
**Fixed effect model**			**0.85**	**[0.84; 0.85]**	**100.0%**	**--**
**Random effects model**			**0.86**	**[0.83; 0.89]**	**--**	**100.0%**

Heterogeneity: $I^2 = 98\%$, $\tau^2 = 0.0214$, $p = 0$

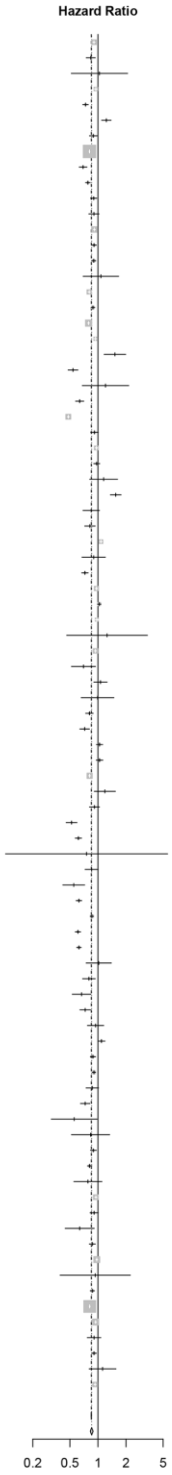

Supplementary Figure 1. Forest plots of the association between gender difference and the risk of cancer patients (Female vs· Male). Each square represents a study, and the size of the square represents the weighting of each study. The diamond box indicates the 95%CI. Heterogeneity is denoted by the I^2 and τ^2· p <0·001 in random effects model. HR= hazard ratio, CI=confidence interval.

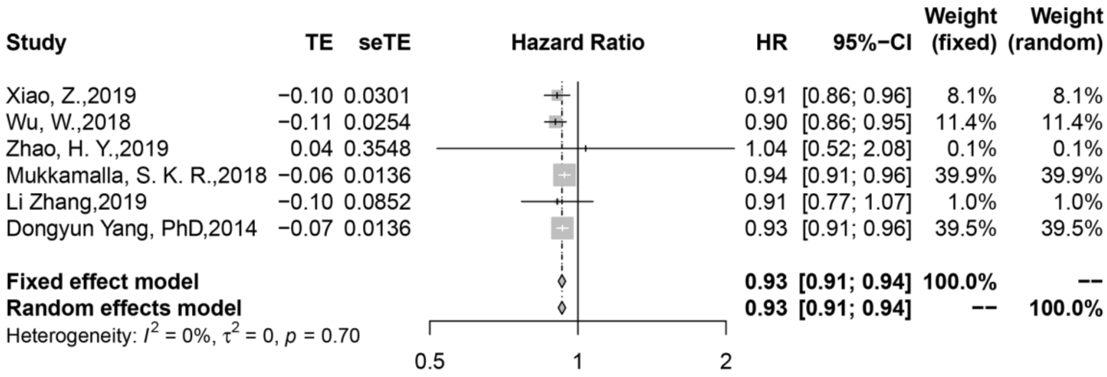

Supplementary Figure 2. Forest plots of the association between gender difference and the risk of liver cancer (Female vs. Male). Each square represents a study, and the size of the square represents the weighting of each study. The diamond box indicates the 95%CI. Heterogeneity is denoted by the I^2 and τ^2. p <0·001 in the fixed effect model. HR= hazard ratio, CI=confidence interval.

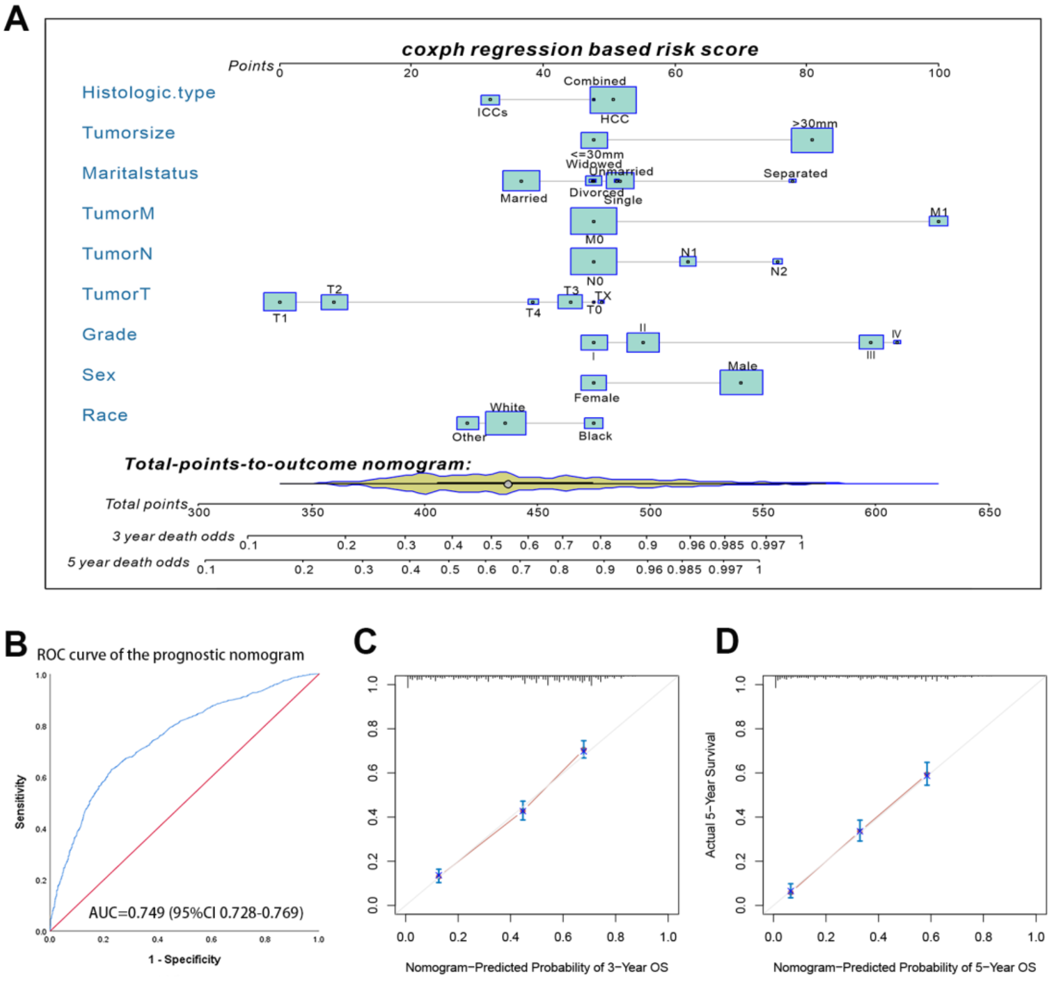

Supplementary Figure 3. Nomogram and ROC curve for non-surgical liver cancer patients before women menopause (established by Cox regression model). (**A**) A survival nomogram for predict 3- and 5-year OS rates of non-surgical liver cancer patients before women menopause (i·e·, ≤ 55 years of age). The yellow Violin Plot and the light blue box display the distribution of patients in the primary cohort. The size of the light blue box represents the proportion of patients. (**B**) Receiver operating characteristic (ROC) curves for prediction models. (**C**, **D**) The calibration curve of the nomogram-predicted probability (3-year OS and 5-year OS, respectively). Age, sex, race, grade, tumor TNM stage, marital status, tumor size, and histological type are contained in the models, p<0·001.

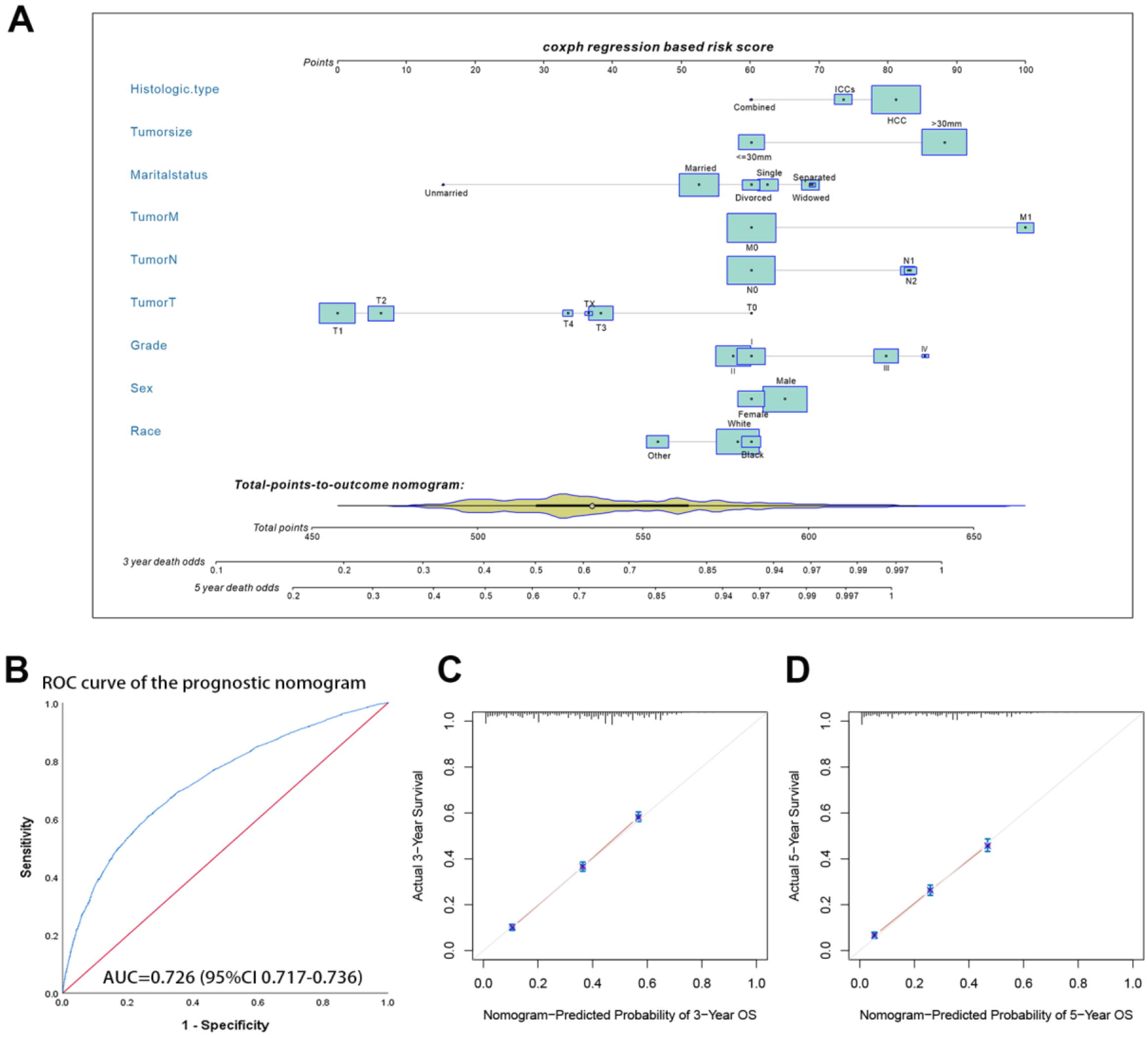

Supplementary Figure 4. Nomogram and ROC curve for non-surgical liver cancer patients after women menopause (established by Cox regression model). (**A**) A survival nomogram for predict 3- and 5-year OS rates of non-surgical liver cancer patients after women menopause (i.e., > 55 years of age). The yellow Violin Plot and the light blue box display the distribution of patients in the primary cohort. The size of light blue box represents the proportion of patients. (**B**) Receiver operating characteristic (ROC) curve for prediction models. (**C**, **D**) The calibration curve of the nomogram-predicted probability (3-year OS and 5-year OS, respectively). Age, sex, race, grade, tumor TNM stage, marital status, tumor size, and histological type are contained in the models, p<0·001.

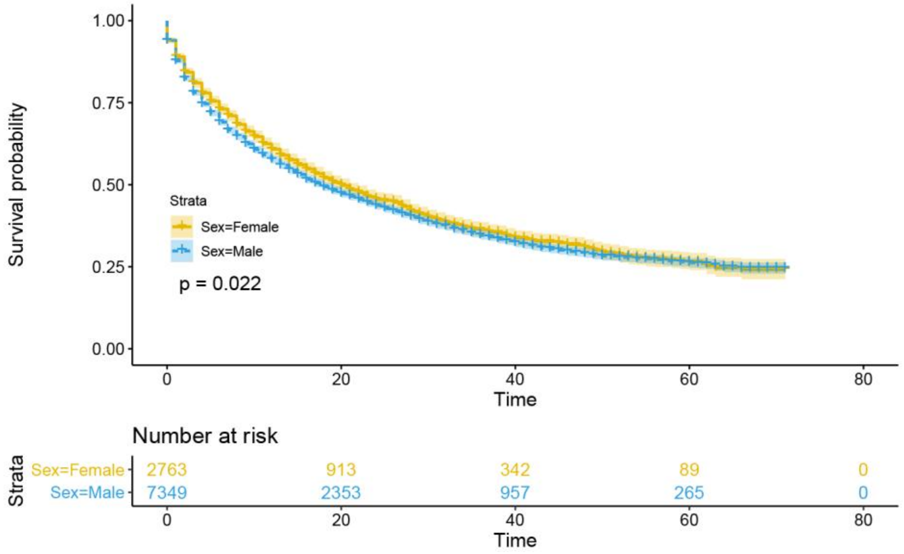

Supplementary Figure 5. Kaplan-Meier survival analysis with log-rank test was performed in non-surgical liver cancer patients after women menopause (i.e. > 55 years of age).
